# Can Ciprofloxacin be Used for Precision Treatment of Gonorrhea in Public STD Clinics? Assessment of Ciprofloxacin Susceptibility and an Opportunity for Point-of-Care Testing

**DOI:** 10.3390/pathogens8040189

**Published:** 2019-10-14

**Authors:** Johan H. Melendez, Yu-Hsiang Hsieh, Mathilda Barnes, Justin Hardick, Elizabeth A. Gilliams, Charlotte A. Gaydos

**Affiliations:** 1 Division of Infectious Diseases, Johns Hopkins School of Medicine, Baltimore, MD 21205, USA; mbarnes2@jhmi.edu (M.B.); jhardic1@jhmi.edu (J.H.); cgaydos@jhmi.edu (C.A.G.); 2 Department of Emergency Medicine, Johns Hopkins University, Baltimore, MD 21205, USA; yhsieh1@jhmi.edu; 3 Johns Hopkins School of Medicine, Baltimore, MD 21205, USA; Elizabeth.Gilliams@baltimorecity.gov

**Keywords:** *Neisseria gonorrhoeae*, gonorrhea, antimicrobial resistance, ciprofloxacin resistance, precision treatment

## Abstract

**Background**: Given the lack of new antimicrobials to treat *Neisseria gonorrhoeae* (NG) infections, reusing previously recommended antimicrobials has been proposed as a strategy to control the spread of multi-drug-resistant NG. We assessed ciprofloxacin susceptibility in a large sample set of NG isolates and identified correlates associated with ciprofloxacin-resistant NG infections. **Methods**: NG isolates collected in Baltimore, Maryland between 2014 and 2016 were evaluated by Gyrase A (*gyrA*) PCR and E-test for susceptibility to ciprofloxacin. Clinical characteristics and demographics were evaluated by multivariate regression analysis to identify correlates of ciprofloxacin-resistant NG infections. **Results**: 510 NG isolates from predominately African American (96.5%), heterosexual (85.7%), and HIV-negative (92.5%) male subjects were included in the study. The overall percentage of isolates with mutant *gyrA* sequences, indicative of ciprofloxacin resistance, was 32.4%, and significantly increased from 24.7% in 2014 to 45.2% in 2016 (p < 0.001). Participants older than 35 years of age were 2.35 times more likely to have a *gyrA* mutant NG infection than younger participants (p < 0.001). Race, sexual orientation, symptomology, or co-infection the HIV or syphilis were not associated with a particular NG *gyrA* genotype. **Conclusions**: Resistance to ciprofloxacin in Baltimore is lower than other regions and indicates that in this environment, use of ciprofloxacin may be appropriate for targeted treatment provided utilization of enhanced surveillance tools. The targeted use of ciprofloxacin may be more beneficial for individuals under 35 years of age. Point-of-care tests for NG diagnosis and susceptibility testing are urgently needed to identify individuals who can be treated with this targeted approach.

## 1. Introduction

Gonorrhea is the second most prevalent bacterial sexually transmitted infection (STI) worldwide, with an estimated 87 million infections in 2016 [1]. In the United States, 555,608 cases of gonorrhea were reported to the Centers for Disease Control and Prevention (CDC) in 2017, a 67% increase from 2013 [2]. *Neisseria gonorrhoeae* (NG) has progressively developed resistance to all commonly-prescribed antimicrobials [3] and is considered as one of the top three urgent threats among antibiotic-resistant bacteria [4]. 

In response to the threat of multidrug-resistant NG, the World Health Organization (WHO) has proposed a global action plan, comprising several strategies, to mitigate the emergence and spread of antimicrobial resistant (AMR) NG [5]. One of the strategies proposed by the WHO to combat AMR NG is the development of molecular methodologies for monitoring and detecting antimicrobial resistance in NG. Traditionally, the determination of AMR NG has been performed by minimum inhibitory concentration (MIC) testing, which requires viable organisms for proper execution. A well developed and validated molecular method for AMR determination could obviate the requirement for a viable clinical isolate. It has been hypothesized that antimicrobial susceptibility testing (AST) at the point-of-care (POC) could lead to precision treatment, i.e., utilizing specific antibiotics based on the AMR profile of NG as opposed to syndromic management of NG infections, including reusing previously recommended antimicrobials. This approach could reduce ceftriaxone selection pressure as ceftriaxone is one of the few remaining antibiotics effective against NG, and help delay the emergence of extended spectrum cephalosporin (ESC) resistance or untreatable gonorrhea [6,7].

Ciprofloxacin, a previously recommended antimicrobial, is an excellent candidate for precision treatment as 69.9% of NG isolates collected by the gonococcal isolate surveillance project (GISP) in 2017 were susceptible to ciprofloxacin [8]. Furthermore, ciprofloxacin susceptibility can be reliably predicted through the detection of genetic markers, thus allowing for the characterization of ciprofloxacin susceptibility directly from a variety of clinical specimens, including those used for the nucleic acid amplification test (NAAT)-based detection of gonorrhea [9]. At the molecular level, resistance to ciprofloxacin is strongly associated with a single mutation at codon 91 of the *gyrase A* (*gyrA*) gene [10], and detection of this mutation has been shown to be an excellent predictor of ciprofloxacin resistance [9,10]. Although other codons in *gyrA*, as well as in topoisomerase IV (*parC*), and in rare instances, penicillin binding protein 2 (PBP2/*PenA*) have been associated with resistance to ciprofloxacin, the vast majority of ciprofloxacin-resistant NG harbor the single mutation at codon 91 of *GyrA*, making this gene the target of choice for molecular screening [3,9]. As such, in 2016, a health care-based system study showed that implementation of a single molecular assay testing for *gyrA* mutations could be utilized in clinical care, and reduced the use of ceftriaxone while increasing the use of ciprofloxacin [11]. Furthermore, patients with wildtype *gyrA* NG who were treated with ciprofloxacin successfully cleared the infection at all anatomical sites of infections [12]. Despite the successful use of ciprofloxacin in clinical settings, this approach has not been evaluated in STI clinic settings, where this approach could potentially be highly effective.

Baltimore, Maryland, a large city with a high prevalence of gonorrhea (691.7/100,000 in 2017) [13], could be an excellent setting for the re-use of ciprofloxacin as a treatment option. According to our previous study, over 55% of the NG isolates collected in 2016 were susceptible to ciprofloxacin [14]. However, additional studies are necessary to better define the epidemiology of ciprofloxacin-resistant NG in Baltimore and determine whether ciprofloxacin could be effectively re-used in this particular population. In this study, we report the epidemiology of ciprofloxacin resistance in Baltimore through the molecular analysis of 510 NG isolates collected between 2014 and 2016. Additionally, clinical characteristics and demographics were evaluated to identify correlates of ciprofloxacin-resistant NG infections. 

## 2. Results

A total of 510 urethral NG isolates collected from 2014 to 2016 were included in the study. The isolates were recovered from 510 male subjects (15–69 years old), predominately African American (96.5%), heterosexual (85.7%), and HIV-uninfected (92.5%) (Table 1). The majority of participants (96.7%) reported symptoms of urethritis at the time of sample of collection, and 4.3% were co-infected with syphilis. 

The number of isolates collected by year was uniformly distributed: 170 (33.3%), 185 (36.3%), and 155 (30.4%), collected in 2014, 2015, and 2016, respectively. Genotypic typing by PCR revealed that 32.4% (165/510) of the isolates had mutation(s) in the *gyrA* gene. The percentage of isolates with *gyrA* mutant sequences was 24.7% (42/170), 28.7% (53/185), and 45.2% (70/155) in 2014, 2015, and 2016, respectively. The increase in the percentage of *gyrA* mutant NG from 2014 to 2016 was statistically significant (p < 0.001) in a bivariate and multivariate regression analysis (Table 2 and Table 3). Multivariate analysis was specifically used to determine the predictor of our outcomes adjusted for co-variates period of the 165 NG isolates with mutant *gyrA* sequences; 63.6% (105/165) were viable for susceptibility testing, and 98.1% (103/105) were confirmed as ciprofloxacin resistant by the E-test method. The remaining two isolates displayed intermediate resistance to ciprofloxacin. Susceptibility testing of 92 randomly selected isolates with *gyrA* wildtype sequences showed that all isolates were susceptible to ciprofloxacin. 

Stratification of the isolates by *gyrA* genotype revealed that older age was associated with a mutant *gyrA* genotype (p = 0.002), suggestive of an association between ciprofloxacin resistance and age (Table 2), and participants ≥35 years of age were 2.35 times (95% CI, 1.47–3.76) more likely to have a *gyrA* mutant NG infection than younger participants (p < 0.001) (Table 3). Race, sexual orientation, symptomology, or co-infection with another STI (HIV or syphilis) were not associated with a particular NG *gyrA* genotype (Table 2). 

## 3. Discussion

As the first step in the development of a POC test for gonorrhea diagnosis and antimicrobial susceptibility testing, we sought to determine whether ciprofloxacin could be a suitable antimicrobial for precision treatment in Baltimore. Using a molecular approach, we have shown low to moderate levels of ciprofloxacin resistance in Baltimore during a three-year period, suggesting that this antimicrobial might be a suitable option for precision treatment. Furthermore, our study identified an association between age and ciprofloxacin-resistant NG infections. 

The overall percentage (32.4%) of mutant *gyrA* NG infections, and thus resistance to ciprofloxacin, in our study is similar to the nationwide percentage (30.1%) reported by GISP in 2017 [8]. The high percentage of wildtype *gyrA* (ciprofloxacin-susceptible) NG isolates in this study provides further evidence for the use of ciprofloxacin for targeted precision treatment, which may help to delay the emergence and spread of resistance to current first-line regimens [6,7]. Additionally, use of ciprofloxacin could make partner therapy easier. However, the increase in the percentage of isolates with mutated *gyrA* sequences from 2014 to 2016 (24.7 to 45.2%), which is consistent with national trends [15], suggests that ciprofloxacin resistance has persisted despite ciprofloxacin not being used as a recommended treatment option for gonorrhea. Therefore, the reintroduction of ciprofloxacin as a gonorrhea treatment option will require enhanced surveillance practices to determine if and when the targeted treatment is no longer a viable option. 

Our study also identified age as an important demographic correlate associated with ciprofloxacin-resistant NG infections. Men older than 35 years of age were 2.35 times more likely to have a mutant *gyrA* NG infection than younger men. On the contrary, men under 24 years of age were more likely to have a wildtype *gyrA* (ciprofloxacin-susceptible) infection. These findings suggest that younger individuals (<24 years of age) may be the ideal population for targeted precision treatment with ciprofloxacin. A targeted treatment approach may prove efficacious in older individuals, but caution is warranted considering the higher percentage of ciprofloxacin resistance observed in this study and the results of previous studies which have reported an association between older age and antimicrobial-resistant NG infections [16]. Contrary to previous studies [16], our study did not find an association between ciprofloxacin-resistant NG and sexual orientation; however, the number of NG isolates from men who have sex with men (MSM) was limited, and a larger study focusing on MSM may provide more details.

The re-introduction of ciprofloxacin as a treatment option could help to mitigate the emergence and spread of AMR NG. According to a modeling study, the introduction of a POC ciprofloxacin susceptibility test could help to decrease the use of ceftriaxone by as much as 66%, thus potentially helping to extend the usefulness of this antimicrobial [17]. However, caution is warranted since the introduction of a POC test targeting a single antimicrobial, such as ciprofloxacin, may accelerate the emergence of triply-resistant (ciprofloxacin, ceftriaxone, and azithromycin) NG isolates [7]. These modelling studies highlight the need for the development of POC tests for resistance to multiple antimicrobials, but the complex resistance mechanisms of other antimicrobials, such as ceftriaxone [3], have hindered the development of such molecular tests. Phenotypic tests, on the other hand, may determine resistance to multiple antimicrobials, but are not currently available at the POC. Therefore, until POC tests targeting multiple antimicrobials can be developed, a single antimicrobial POC test, capable of providing an alternative treatment option, such as ciprofloxacin, might be the most suitable option. The development of a POC test for gonorrhea and identification of ciprofloxacin susceptibility is attractive because ciprofloxacin can be administered before the patient leaves the clinic.

Our study had several limitations. First, all of the isolates were collected in Baltimore, Maryland at one clinic, thus limiting the generalizability and scope of these results. However, the data reported by GISP in 2017 suggest low levels of ciprofloxacin resistance in the US [8]. Second, we had limited access to epidemiological, complete demographic, and behavioral data, which limited the scope of our analysis. It should also be noted that these samples were collected between 2014 and 2016; it is likely that the levels of ciprofloxacin susceptibility and resistance have varied since then, and these results should be viewed with that caveat. Finally, samples from women and samples from extragenital sites (pharyngeal and rectal) were not available for this analysis. Additionally, a more complete approach to the study would have been to expand molecular testing to include *ParC* targets associated with ciprofloxacin resistance, as well as the rare *PenA* target associated with ciprofloxacin resistance. However, as stated previously, the vast majority of ciprofloxacin-resistant NG harbor the single mutation at codon 91 of *GyrA*, and focusing on this target, as similar studies have shown, proves to be just as effective at identifying ciprofloxacin susceptibility and resistance, as expanded tests show.

## 4. Conclusions

In conclusion, we have shown that a large proportion of the NG isolates tested in this study are susceptible to ciprofloxacin, providing further support for the use of this antimicrobial for targeted treatment if ciprofloxacin susceptibility can be determined at the POC. Current treatment guidelines for NG from multiple health organizations state that ceftriaxone (CRO) plus azithromycin (AZM), or cefixime (CFX), are the standards, but these guidelines are based on a syndromic approach to disease management [3,5]. Given the overall increase in resistance to multiple classes of antibiotics, syndromic management of NG may no longer be the most appropriate strategy. Even in this study, the increase in resistance during the study period highlights the need for continued and improved surveillance practices. Given the lower proportion of ciprofloxacin resistance in younger individuals, a targeted surveillance approach may be more beneficial for patients under 35 years of age as it increases the likelihood of identifying ciprofloxacin susceptible NG. Additional studies aimed at identifying facilitators and potential barriers towards the implementation of POC susceptibility testing and precision treatment in the STI clinic setting are warranted. 

## 5. Materials and Methods

### 5.1. Clinical Data

This study was approved by the Johns Hopkins University School of Medicine Institutional Review Board. For purposes of statistical analysis to determine potential risk factors and clinical correlates associated with acquisition of ciprofloxacin-resistant NG, clinical data (age, race, sexual orientation, HIV status, current and past diagnosis of a Syphilis infection) were collected by record review.

### 5.2. NG Isolates

NG isolates were recovered from the urethra of symptomatic men seeking STI testing at the Baltimore City Health Department (BCHD) Druid Health Clinic from January 2014 to October 2016. Following culture, gram-negative diplococci, oxidase-positive bacterial isolates were presumptively classified as NG, stored in trypticase soy broth (TSB) with 20% glycerol, and frozen at −80 °C.

For this study, isolates were recovered by growth on chocolate agar plates incubated overnight in 5% CO_2_ and the colonies re-suspended in phosphate buffered saline (PBS). Two-hundred microliters of each bacterial suspension was extracted for DNA using the automated MagNA Pure LC instrument (Roche Diagnostics, Indianapolis, IN, USA). Following DNA extraction, the isolates were confirmed as NG using a previously described PCR assay targeting the *opa* gene [18,19]. For non-viable isolates, 200 µL of the isolate-containing TSB media was extracted for total DNA as described above. 

### 5.3. Molecular Characterization of Ciprofloxacin Susceptibility 

In order to determine ciprofloxacin susceptibility at the molecular level, all isolates (viable and non-viable) were analyzed by real-time PCR for the presence/absence of mutation(s) in the *gyrA gene* using a previously described assay [19,20]. The PCR assay targets wildtype *gyrA* sequences, which are highly predictive of ciprofloxacin susceptibility [9]. NG isolates with negative *gyrA* PCR results were classified as *gyrA* mutant, which is indicative of ciprofloxacin resistance.

### 5.4. Antimicrobial Susceptibility Testing

All viable NG isolates with mutated *gyrA* sequences were further analyzed by the E-test method, (bioMérieux, Durham, NC, USA), to determine the minimum inhibitory concentration (MIC) of ciprofloxacin, as we previously described [14]. Additionally, all viable NG isolates with wildtype *gyrA* sequences, collected in 2016, and a subset of isolates collected in 2014 were also tested using the E-test method. Phenotypic susceptibility testing on isolates collected in 2015 was not performed, because none of those isolates were viable for analysis. Briefly, bacterial suspensions, matching a 0.5 MacFarland standard, were plated on GC agar medium (Becton Dickinson, Sparks, MD, USA) media supplemented with 1% IsoVitaleX (Becton Dickinson, Franklin Lakes, NJ, USA) and allowed to air dry for 5 minutes. E-test strips containing ciprofloxacin were individually placed on the inoculated agar surface according to manufacturer’s recommendations, incubated at 37 °C in a moist 5% CO_2_-enriched environment, and MIC results recorded after 18–24 h. The MIC was determined by reading the intercept of the inhibition zone and the E-test strip. Breakpoints for ciprofloxacin resistance were selected in accordance with guidelines set forth by the Clinical and Laboratory Standards Institute (CLSI) [21]. 

### 5.5. Statistical Analysis

Bivariate analysis was performed using the chi-square test. The Cochran–Armitage Trend test was performed to evaluate yearly trends. The multivariate model was used to determine the predictor of our outcomes adjusted for co-variates. In this case, age group was the key predictor, and we strongly believed that calendar years and others could be either covariates for the outcomes. Therefore, we built this full model first, then used multivariate analysis to identify the final model. The final model was built in a stepwise manner. All analyses were conducted using SAS version 9.4 

## Figures and Tables

**Table 1:** Characteristics of 510 men with *Neisseria gonorrhoeae* infection in Baltimore, 2014–2016.

Characteristics	Categories	Number (%)
		N = 510
Age (Years)	15–19	60 (11.8)
	20–24	117 (22.9)
	25–29	109 (21.4)
	30–34	57 (11.2)
	35–44	85 (16.7)
	≥45	82 (16.1)
Race/Ethnicity	African American	492 (96.5)
	Non-Hispanic White	11 (2.2)
	Hispanic	3 (0.6)
	Other	4 (0.8)
Sexual Orientation	Heterosexual	437 (85.7)
	Bisexual	17 (3.3)
	Gay	50 (9.8)
	Unknown/Unspecified	6 (1.2)
Calendar Year	2014	170 (33.3)
	2015	185 (36.3)
	2016	155 (30.4)
Symptoms	Discharge	459 (90.0)
	Dysuria	253 (49.6)
	Itch in urogenital area	19 (3.7)
	Lesion in urogenital area	16 (3.1)
	Irritation or tingling feeling	13 (2.5)
	Burning sensation	7 (1.4)
	Rash	6 (1.2)
	Pain in urogenital area	5 (1.0)
	Other	2 (0.4)
	None	17 (3.3)
HIV Infection	Yes	34 (6.7)
	No	472 (92.5)
	Unknown	4 (0.8)
Concurrent Syphilis Infection	Yes	22 (4.3)
	No	488 (95.7)
Syphilis Diagnosis in the Past	Yes	25 (4.9)
	No	485 (95.1)
*GyrA* Genotype	Wild type	345 (67.7)
	Mutant	165 (32.4)

**Table 2:** Bivariate analysis of association between demographic and clinical characteristics and *gyrA* genotype among 510 men with *Neisseria gonorrhoeae* infection in Baltimore, 2014–2016.

		*gyrA* Genotype		p-Value
Characteristics	Categories	Wild Type	Mutant	
		N = 345	N = 165	
Age (Years)*	15–24	135 (39.1)	42 (25.5)	0.002
	25–34	113 (32.8)	53 (32.1)	
	≥35	97 (28.1)	70 (42.4)	
Race/Ethnicity	African American	334 (96.8)	158 (95.8)	0.546
Sexual Orientation	Bisexual or Gay	51 (14.8)	16 (9.7)	0.112
Calendar Year†	2014	128 (37.1)	42 (25.5)	<0.001
	2015	132 (38.3)	53 (32.1)	
	2016	85 (24.6)	70 (42.4)	
Symptom—Discharge	Discharge	312 (90.4)	147 (89.1)	0.636
Symptom—Dysuria	Dysuria	176 (51.0)	77 (46.7)	0.358
No Symptom	Yes	9 (2.6)	8 (4.9)	0.187
HIV Infection	Yes	26 (7.5)	8 (4.9)	0.255
Concurrent Syphilis Infection	Yes	15 (4.4)	7 (4.2)	0.956
Syphilis Diagnosis in the Past	Yes	16 (4.6)	9 (5.5)	0.689

* p < 0.001 for Cochran–Armitage Trend Test; † p < 0.001 for Cochran–Armitage Trend Test.

**Table 3:** Multivariate regression analysis of factors associated with presence of *gyrA* genotype among 510 men with *Neisseria gonorrhoeae* infection in Baltimore, 2014–2016.

Variables	Categories	Odds Ratio (95% CI)	p-Value
Age Group (years)	15–24	1.00	
	25–34	1.46 (0.90, 2.37)	0.123
	≥35	2.35 (1.47, 3.76)	<0.001
Calendar Year	Increasing each year	1.61 (1.27, 2.05)	<0.001
